# The complete mitochondrial genome sequence of *Populus davidiana* Dode

**DOI:** 10.1080/23802359.2017.1289346

**Published:** 2017-02-16

**Authors:** Mi Na Choi, Muho Han, Hyoshin Lee, Hong-Seog Park, Min-Young Kim, Ji-Seon Kim, Yoon-Jeong Na, Seung-Woo Sim, Eung-Jun Park

**Affiliations:** a Division of Forest Biotechnology, National Institute of Forest Science, Suwon, Republic of Korea;; b GnC Bio Co., Yekun-plaza, Daejeon, Republic of Korea

**Keywords:** Mitochondrial genome, *Populus davidiana* Dode, phylogenetic analysis

## Abstract

This study sequenced the entire mitochondrial genome of *Populus davidiana* Dode. It was 779,361 bp in length, containing 33 protein-coding genes, 3 rRNA genes and 22 tRNA genes and 1 pseudogene, and its GC content was 44.8%. Phylogenetic analysis was conducted using 6 mitochondrial genomes from the *Salicaceae* and *Euphorbiaceae* families, resulting that *P. davidiana* Dode was closely related to *Populus tremula* and *Populus tremula* ×* Populus alba*. These results will provide fundamental data for the evolutionary studies in *Populus* genus.

*Populus davidiana* Dode is mainly distributed in the northeastern hemisphere. It belongs to the subsection trepidae of the section leuce, along with *Populus tremula* L. (European aspen), *P. tremuloides* Michx. (Quaking aspen) and *P. grandiden* (big-tooth aspen) (Lee et al. 2011). This species has been extensively planted in Korea for biomass production in short rotation plantations. In the genus *populus*, only two complete mitogenomes have been sequenced available for *P. tremula* (Genbank: KT337313) and *Populus tremula* ×* Populus alba* (Genbank: KT429213) (Kersten et al. 2016). In this study, we provided the complete mitochondrial genome sequence (Genbank: KY216145) of *P. davidiana* Dode and compared its mitogenome sequences with those of six others available from the *Salicaceae* and *Euphorbiaceae* families.

The plant material of the female *P. davidiana* (Accession number, Odae 19) was collected from Kyungbuk-Youngju clonal plantation (36°49´N 128°31´E) in Republic of Korea, which was the same plant material previously used for the chloroplast genome sequencing (Choi et al. 2016). Total genomic DNA was extracted from fresh leaves using the DNeasy Plant Mini Kit (Qiagen, Valencia, CA). The three independent sequencing libraries were prepared; shotgun library for 454 GS FLX and 3kb and size free mate-pair libraries for Illumina Hiseq 2500. A total of 1021M raw reads were retrieved and quality-trimmed with default parameters using SOAPec v2.03 (Beijing Genomics Institute, Shenzhen, China), Cutadapt (Department of Computer Science, TU Dortmund, Science for Life Laboratory), DeconSeq-standalone v0.4.3 and Sickle v1.33 (GitHub, Inc., San Francisco, CA). The resultant 935M trimmed reads were then used for the mitochondrial genome reconstruction using the PLATANUS assembler, by mapping them to the reference mitogenome of *P. tremula*. A total of 14,136,698 individual mitochondrial reads generated up to 2228 × average coverage. Subsequently the mitochondrial genome of *P. davidiana* was annotated using BLASTn and ORF Finder at NCBI by comparing with that of *P. tremula*.

The complete mitochondrial genome of *P. densiflora* is 779,361bp in length, which is smaller than *P. tremula* (783,442bp) and *P. tremula* ×* P. alba* (783,513bp). The mitochondrial genome contains 59 genes, including 33 protein-coding genes, 3 ribosomal RNA genes, 22 transfer RNA genes and one pseudogene (*rpl16*). Among 33 protein-coding genes (a total of 57,067bp in length), the largest gene was *nad4* (8370 bp) while *atp9* was the smallest gene (224 bp). The GC content of the mitochondrial genome is 44.8%.

The complete mitochondrial genome of *P. davidiana* was aligned with those of 6 other species available from the *Salicaceae* and *Euphorbiaceae* families. Based on this alignment, a phylogenetic tree was generated by UPGMA method of MEGA 7 using 1000 bootstrap replicates (Kumar et al. 2016) (Figure 1). Branches corresponding to partitions reproduced in less than 50% bootstrap replicates are collapsed. The percentage of replicate trees in which the associated taxa clustered together in the bootstrap test is shown next to the branches (Felsenstein 1985). The evolutionary distances were computed by Maximum Composite Likelihood (MCL) method and are in the units of the number of base substitutions per site (Tamura et al. 2004). All *Populous* mtDNA sequences clustered together placing other three genera, *Salix*, *Hevea*, and *Ricinus*, as outgroups. The *P. davidiana* mtDNA sequence was very similar to both *P. tremula* and *P. tremula* ×* P. alba* with the same nucleotide identity rate (99.0%).

**Figure 1.**
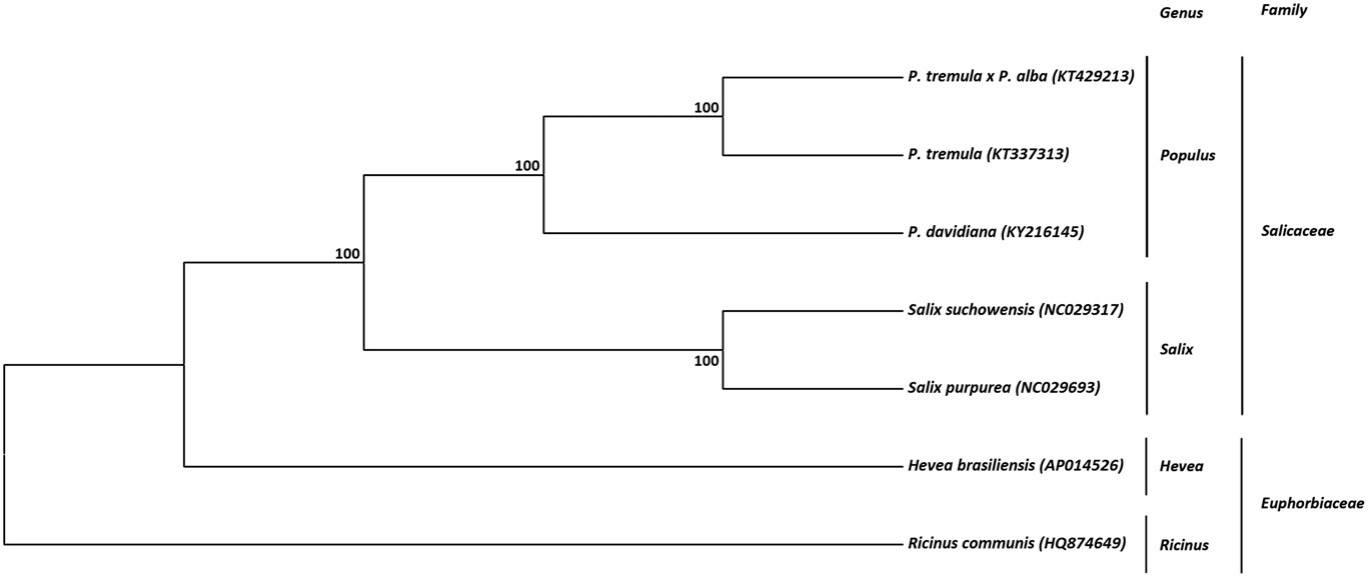
Phylogenetic tree based on seven complete mitochondrial genome sequences from the *Salicaceae* and *Euphorbiaceae* families. The tree was constructed using UPGMA method and bootstrap support values (%) from 1000 replicates are shown above branches. GenBank accession numbers: *Populus davidiana* (KY216145), *Populus tremula* (KT337313), *Populus tremula x Populus alba* (KT429213), *Salix suchowensis* (NC029317), *Salix purpurea* (NC029693), *Hevea brasiliensis* (AP014526), *Ricinus communis* (HQ874649).
